# Emergence of Crimean–Congo Hemorrhagic Fever Virus in Eastern Senegal in 2022

**DOI:** 10.3390/v16020315

**Published:** 2024-02-19

**Authors:** Ousseynou Sene, Samba Niang Sagne, Déthié Ngom, Moussa Moise Diagne, Aminata Badji, Aliou Khoulé, El Hadji Ndiaye, Safietou Sankhe, Cheikh Loucoubar, Mawlouth Diallo, Manfred Weidmann, Ndongo Dia, Etienne Simon-Lorière, Yoro Sall, Boly Diop, Mamadou Ndiaye, Anavaj Sakuntabhai, Amadou Alpha Sall, Ousmane Faye, Oumar Faye, Diawo Diallo, Mamadou Aliou Barry, Gamou Fall

**Affiliations:** 1 WHO Collaborating Centre for Arbovirus and Viral Hemorrhagic Fevers, Virology Department, Pasteur Institute, Dakar 12900, Senegal; ousseynou.sene@pasteur.sn (O.S.); moussamoise.diagne@pasteur.sn (M.M.D.); safietou.sankhe@pasteur.sn (S.S.); ndongo.dia@pasteur.sn (N.D.); amadou.sall@pasteur.sn (A.A.S.); ousmane.faye@pasteur.sn (O.F.); oumar.faye@pasteur.sn (O.F.); 2 Epidemiology, Clinical Research & Data Science, Pasteur Institute, Dakar 12900, Senegal; sambaniang.sagne@pasteur.sn (S.N.S.); cheikh.loucoubar@pasteur.sn (C.L.); aliou.barry@pasteur.sn (M.A.B.); 3 Zoology Department, Pasteur Institute, Dakar 12900, Senegal; dethie.ngom@pasteur.sn (D.N.); aminassbadji@gmail.com (A.B.); aliou.khoule@pasteur.sn (A.K.); elhadji.ndiaye@pasteur.sn (E.H.N.); mawlouth.diallo@pasteur.sn (M.D.); diawo.diallo@pasteur.sn (D.D.); 4 Institute of Microbiology and Virology, Brandenburg Medical School, 01968 Brandenburg, Germany; manfred.weidmann@mhb-fontane.de; 5 G5 Evolutionary Genomics of RNA Viruses, Pasteur Institute, 75015 Paris, France; etienne.simon-loriere@pasteur.fr; 6 Ministry of Health, Dakar 10700, Senegal; yorosall2005@yahoo.fr (Y.S.); diopboly@yahoo.fr (B.D.); mamamorph@yahoo.fr (M.N.); 7 Functional Genetics of Infectious Disease Unit, Pasteur Institute, 75015 Paris, France; anavaj.sakuntabhai@pasteur.fr; 8 Centre National de la Recherche Scientifique (CNRS), UMR2000, Department of Global Health, 75015 Paris, France; 9 International Vaccine Design Center (vDesC), The Institute of Medical Science, The University of Tokyo (IMSUT), Tokyo 108-8639, Japan

**Keywords:** Crimean–Congo hemorrhagic fever virus, human, ticks, reassortment, Eastern Senegal

## Abstract

Crimean–Congo hemorrhagic fever (CCHF), the most widespread tick-borne viral human infection, poses a threat to global health. In this study, clinical samples collected through national surveillance systems were screened for acute CCHF virus (CCHFV) infection using RT-PCR and for exposure using ELISA. For any CCHF-positive sample, livestock and tick samples were also collected in the neighborhood of the confirmed case and tested using ELISA and RT-PCR, respectively. Genome sequencing and phylogenetic analyses were also performed on samples with positive RT-PCR results. In Eastern Senegal, two human cases and one *Hyalomma* tick positive for CCHF were identified and a seroprevalence in livestock ranging from 9.33% to 45.26% was detected. Phylogenetic analyses revealed that the human strain belonged to genotype I based on the available L segment. However, the tick strain showed a reassortant profile, with the L and M segments belonging to genotype I and the S segment belonging to genotype III. Our data also showed that our strains clustered with strains isolated in different countries, including Mauritania. Therefore, our findings confirmed the high genetic variability inside the CCHF genotypes and their introduction to Senegal from other countries. They also indicate an increasing CCHF threat in Senegal and emphasize the need to reinforce surveillance using a one-health approach.

## 1. Introduction

Crimean–Congo hemorrhagic fever (CCHF) is an emerging anthropo-zoonosis occurring in Africa, Asia, and Europe [1]. The name of the disease derives from the regions where it was first reported (Crimea (1945) and Congo (1956)) [2]. The disease is caused by a virus (CCHFV) belonging to the genus *Orthonairovirus* of the family *Nairoviridae* in the order *Bunyavirales* [3]. The zoonotic life cycle of the CCHFV includes ticks as vectors and reservoirs, and animals as amplifying hosts or dispersal vectors [1]. Humans can be infected by tick bites, mainly of the genus *Hyalomma*; by contact with a CCHF patient during the acute phase of infection; or by contact with the blood or tissues of viremic livestock [4]. CCHF patients present with a wide range of clinical symptoms characterized by the sudden onset of fever, muscle pain, headache, dizziness, photophobia, and hyperemia. In severe cases, hemorrhagic manifestations develop several days after the onset of the disease [5]. The mean of the annual and periodic CCHF fatality rates indicates that fatalities occur in about one tenth of human CCHF cases [6]. This fatality rate can increase to 50% in hospitalized patients [7].

The CCHFV is an enveloped virus with three negative-sense genome segments: short (S), medium (M), and long (L) [5]. The virus has several distinct lineages, also called genotypes (Gt) [8]. Several classifications have been proposed, and the strains of the CCHFV are currently classified into five genotypes: I–III (endemic in Africa), IV (in Asia), and V (in Europe) [9,10]. Genotype VI no longer exists based on a new classification where the CCHF strains that belonged to genotype IV have been reclassified to the Aigai viral species, which rarely cause severe illness [10].

The geographical range of the CCHFV is the widest among tick-borne viruses of human health concern and the second widest, after dengue, among medically important arboviruses [1]. CCHF epidemics have been documented in Africa, the Middle East, Eastern Europe, and Central and Western Asia [5]. CCHF epidemics pose a threat to global health because of their epidemic potential, high mortality rate, potential for nosocomial infection, and difficulties in treatment and prevention [9].

In West Africa, the first human case of CCHF was detected in Mauritania in 1983 [11]. Since then, numerous outbreaks of CCHF have been reported in Mauritania, while only a few sporadic cases have been detected in humans in Senegal [12,13]. Previous studies have shown the circulation of this virus in ruminants and ticks, with a high prevalence in Northern Senegal at the border with Mauritania, where several strains of the CCHFV have been isolated [14]. Recently, a survey conducted in the Matam region (Northeast Senegal) reported high seroprevalence in livestock animals and one sporadic human case and described strains from ticks belonging to different genotypes [13]. All these data indicate that there is a high risk of CCHF emergence in Northern Senegal.

Given this high emergence risk in Northern Senegal and the existence of several CCHF genotypes, reliable and up-to-date surveillance systems are crucial to better evaluate the incidence of the CCHFV and its spread in other Senegalese regions. Here, we describe two CCHF cases detected in Eastern Senegal in 2022: one in Matam (Northeast Senegal) detected through sentinel syndromic surveillance and one in Tambacounda (Southeast Senegal) detected through viral hemorrhagic fever surveillance in hospitals. We also describe the detection of the CCHV in *Hyalomma* ticks from Tambacounda and the animal exposure in both areas and present the molecular characterization of the strains identified in humans and ticks.

## 2. Materials and Methods

### 2.1. Study Area

CCHF was detected in Koumpentoum and Bokidiawe, which are located, respectively, in the regions of Tambacounda (13°46′08″ north, 13°40′02″ west) and Matam (15°06′ north, 13°38′ west). These two regions in Eastern Senegal are bordered by the Senegal river for approximately 200 km and the Islamic Republic of Mauritania.

The Tambacounda region is located in the sparsely populated Sahelian plains of Eastern Senegal. Tambacounda has a tropical savannah climate with two seasons: a rainy season from June to October, characterized by heat, humidity and storms, and a sweltering dry season without rain from November to May. The average annual precipitation is 887 mm. The Tambacounda region is in a tall-grass and acacia savanna area known for its varied agricultural potential. The Tambacounda region also hosts a national zoological park with very rich wildlife. Local activities include cattle ranching, cotton farming, and tourism.

The Matam region is an important agro-sylvo-pastoral zone. Matam has a hot semi-arid climate marked by the presence of the harmattan in the dry season (November to June) and monsoon winds in the rainy season (July to October). The average annual precipitation is 369 mm. The area is home to an important hydrographic network and major hydrogeological resources characterized by the presence of numerous ponds and backwaters fed by rainwater, which serve multiple functions, mainly the watering of livestock. The area is covered with vegetation dominated by *Acacia nilotica*, *Balanites*, and *Zizyphus*. Agriculture and livestock breeding are the main socio-professional activities of the habitants.

### 2.2. Human Serum Collection

We used two surveillance systems for the detection of the CCHF cases: the viral hemorrhagic fever (VHF) surveillance focused on patients with fever associated with hemorrhagic symptoms in regional hospitals and the sentinel syndromic surveillance of Senegal (4S network), which is implemented in health centers or posts in all regions of Senegal. The syndromic surveillance system is based on fever associated with at least two of the following signs: headache, arthralgia, myalgia, rash, retro-orbital pain, encephalitis, and hemorrhagic symptoms. This 4S system usually allows the early detection of viruses in Senegal [15,16]. For all patients presenting the clinical case definitions of these two surveillance systems, a blood sample is collected and shipped to the WHO Collaborating Center for Arboviruses and VHF at Institut Pasteur de Dakar (IPD) for laboratory diagnosis.

### 2.3. Animal Sample Collection

Blood samples were collected from sheep and goats in the neighborhoods of the confirmed human cases. The samples were collected (approximately 5 mL) from the jugular veins of animals using Vacutainer tubes (venipuncture) by local veterinarians. After centrifugation at 5000× *g* for 10 min, the serum was aliquoted in 2 mL sterile Nunc tubes (Nunc, Denmark) and sored at −80 °C for analysis.

### 2.4. Tick Collection

Ticks were collected during outbreak investigations in Koumpentoum and Bokidiawe using forceps from randomly selected livestock (sheep, cattle, and goats) in the houses and neighborhoods of the confirmed cases. The collected ticks were morphologically identified on a chill table based on available taxonomic keys [17,18]. They were then grouped into pools (by species, sex, and animal source) and stored at −80 °C for analysis.

### 2.5. Laboratory Tests

The clinical samples were tested using in-house IgM ELISA assays for different viruses, including CCHF, using antigens and immune ascites produced in mice at IPD, as previously described [15]. RNA was also extracted from the clinical samples using a QIAamp viral RNA mini kit according to the manufacturer’s instructions (Qiagen, Hilden, Germany). RT-PCRs were performed for the Dengue virus, Yellow fever virus, Chikungunya virus, West Nile virus, Zika virus, Rift Valley fever virus, and CCHFV [15]. RT-PCR for the CCHFV was performed as previously described [19]. As we do not have an official testing algorithm for CCHF and information regarding the date of symptom onset can be missing in some case investigation forms, all the samples collected from the surveillance systems were screened using RT-PCR and IgM ELISA to avoid case misdiagnosis.

Regarding the animal samples, they were tested using RT-PCR as described above for human samples, and the sheep and goat samples were also tested using IgG ELISA according to a previously described method for working with samples from these 2 species [20]. Briefly, CCHF recombinant glycoprotein (Gn, from Sinobiological) was used to coat the plates, and secondary antibodies coupled with horseradish peroxidase (rabbit anti-sheep/goat IgG from Biorad) were also used. Optical densities were read using 450/620 filters, and the cutoff was determined using a finite mixture model in R software version 4.3.2 [20].

The tick pools were each placed in a 2 mL tube containing 3 metal beads and 1 mL of a cell culture medium consisting of L-15 (Gibco BRL, Grand Island, NY, USA) and 20% cold fetal serum (FBS). This mixture was automatically ground using a Tissue Lyser (Qiagen) for 2 min at a frequency of 30 hertz/second and then centrifuged at 10,000 rpm at +4 °C for 5 min. After centrifugation, the supernatant was filtered using a 0.20 µm filter (Sartorius, Göttingen, Germany), collected in a CryoTube (Thermo Scientific, Waltham, MA, USA) using a 1 mL syringe (Artsana, Como, Italy), and stored at −80 °C. Subsequently, 140 µL was used for each tick sample for RNA extraction using a QIAamp Viral RNA mini kit, and CCHF RT-PCR was conducted using the method previously described.

### 2.6. Viral Genome Sequencing and Phylogenetic Analyses

Sequencing was performed according to the Illumina RNA prep with enrichment (L) kit reference guide (1000000124435 v01) on an Illumina MiSeq 150 cycle system. The sequencing reads were analyzed using the EDGE bioinformatics pipeline (https://www.edgebioinformatics.org/, accessed on 5 January 2022), which generated a single Fasta file for each sample. Nearly complete genomes obtained during this work were submitted to a public nucleotide BLAST database to identify their homologous sequences. They were then combined with a representative subset of CCHFV sequences available in GenBank.

Sequences were aligned using the MAFFT program [21]. Maximum-likelihood (ML) phylogenetic trees were inferred using IQ-Tree software version 1.6.0, adding an automatic model selection argument using model finder (MF) as implemented in the software using the Bayesian Information Criterion, and were tested using a bootstrap method with 1000 replicates [22]. The trees were visualized and annotated using Figtree [23]. Similarity plot (SIMPLOT) v. 3.5.1 was used to further analyze the genetic variations in the S, M, and L segments between our new CCHF strains and other strains circulating in Senegal, Mauritania, Spain, and Asia [24].

## 3. Results

### 3.1. Human Case Presentation

- Detection of one Human case in Koumpentoum, Tambacounda region

**Figure 1:**
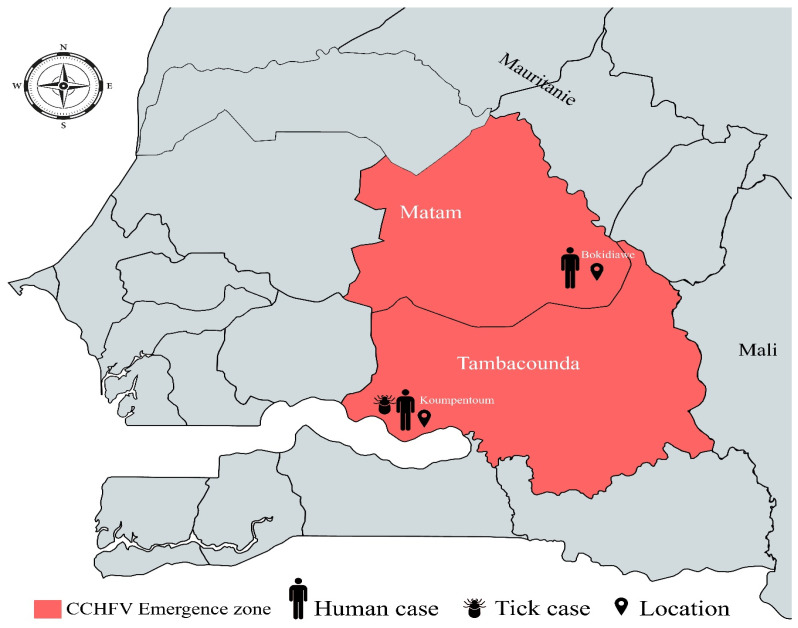
Regions and locations of Crimean–Congo hemorrhagic fever virus detection in humans and ticks in 2022 in Eastern Senegal.

On 15 February 2022, a 40-year-old woman from the Koumpentoum district (Tambacounda region) (Figure 1) was admitted to the regional hospital due to a fever (T = 39 °C) accompanied by muscle and joint pains. She also exhibited hemorrhagic symptoms including gingivorrhagia, hematemesis, metrorrhagia, epistaxis, and rectorrhagia. The illness began on 6 February 2022, with initial symptoms of headaches, joint pains, and a vesperal fever without chills or sweats. Before her admission to this regional hospital, the patient sought medical care at the Velingara Koto health post on 9 February 2022 and the Maleme Niani health post on 10 February 2022. During these visits, she received treatment with antibiotic and fever-reducing medications. As her condition did not improve and her bleeding symptoms worsened (hematemesis, gingivorrhagia, and metrorrhagia), she returned to the Maleme Niani health post. Subsequently, on 12 February 2022, she was referred to the Koumpentoum district health center for more comprehensive care. Laboratory tests conducted at the health center revealed pancytopenia (Hb = 8.3 g/dL, platelets = 110,000, WBC = 3000). As a result, an emergency treatment involving antibiotics, antipyretics, and antihemorrhagics was initiated. Due to a lack of improvement and the development of additional bleeding symptoms (epistaxis and rectal bleeding), along with the appearance of subicterus, the patient was referred to the Regional Hospital of Tambacounda on 14 February 2022. Laboratory tests upon admission revealed severe anemia (BH = 5 g/dL, WBC = 5600) and severe thrombocytopenia (platelets = 36,000). Given these findings, there was a suspicion of a hemorrhagic fever, prompting a blood sample to be sent to IPD on 15 February as part of the routine surveillance for hemorrhagic fevers in Senegal. The sample tested positive for the CCHFV using IgM ELISA but was negative using RT-PCR. The patient was treated with antibiotics, antipyretics, and antihemorrhagics and received multivitamins as well as a blood transfusion. Fortunately, the patient’s condition improved without complications and they were discharged on 21 February 2022.

- Detection of one Human case in Bokidiawe, Matam region

On 17 May 2022, an 84-year-old woman residing in Kirire 2 (Matam region) (Figure 1) presented at the Bokidiawe sentinel site health post with symptoms of fever, headache, and myalgia but without any signs of bleeding. The onset of the illness occurred on 15 May 2022. With a negative malaria RDT result and 4S inclusion criteria, a blood sample was collected on the same day as the medical examination and sent to IPD for laboratory diagnosis. The sample tested positive for CCHF using RT-PCR but was negative using IgM ELISA. The patient was treated with antibiotics and antipyretics. They were followed as an outpatient and seen again 48 h later for evaluation. The patient recovered favorably, and a second blood sample was taken 15 days later. Virological testing of this sample showed seroconversion and the presence of CCHFV IgM antibodies, while RT-PCR yielded a negative result. The patient recovered without complications or sequelae.

### 3.2. Detection of CCHFV IgG Antibodies in Animals

All sheep and goat samples were negative for CCHF using RT-PCR. However, global CCHF seroprevalence values of 45.26% and 9.33% were observed in Bokidiawe and Koumpentoum, respectively (Table 1). In Bokidiawe, the seroprevalence was higher in goats, while in Koumpentoum higher seroprevalence was observed in sheep.

### 3.3. Viral Detection in Ticks

A total of 1055 ticks, grouped into 411 pools according to species, were collected in Koumpentoum, with a predominance of *Hyalomma marginatum rufipes* and *H. truncatum species* (32.6% and 12.7% of the tick fauna, respectively). In Bokidiawe, only 99 ticks, organized in 16 pools, were collected, with a predominance of *H. impeltatum* (74.7%) (Table 2). A single pool containing four *H. m. rufipes* specimens from Koumpentoum was found to be positive for the CCHFV, while all tick pools from Bokidiawe were negative.

### 3.4. Phylogenetic Analyses

In the present study, we assigned the names Boki_2022 and Koum_2022 to the two identified CCHFV strains, denoting the collection year and the collection sites, Bokidiawe and Koumpentoum, respectively. We obtained all segments (S, M, and L) for the tick strain Koum_2022 but only a partial sequence of the L segment for the human strain Boki_2022. BLASTn analyses revealed that our two strains shared very high nucleotide sequence similarity (more than 99%) with strains isolated from Mauritania in 1984 (ABB30015 and ABB30041), Spain in 2014 (ASV45880 and ASV45882), and Nigeria in 1966 and 1996 (AAY24690 and ARB51456).

**Figure 2:**
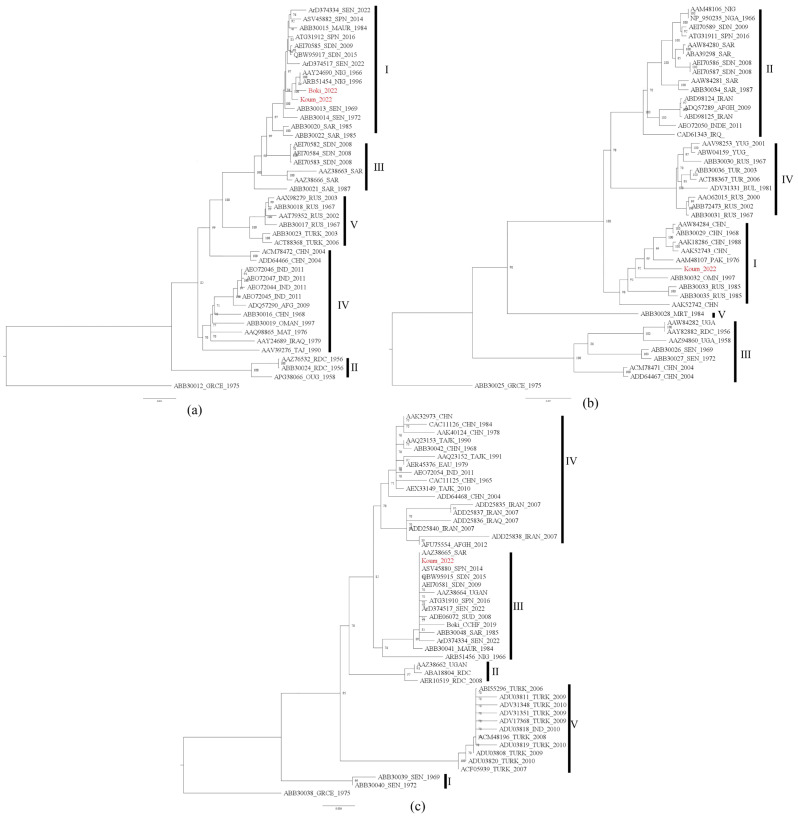
Maximum-likelihood trees of CCHFV strains based on the L (**a**), M (**b**), and S (**c**) segments. L, M, and S segments follow the genotype names described by Papa et al. [10]. Genotypes are identified by Roman numerals. All the newly characterized CCHF isolates from Senegal in 2022 are color-coded in red.

Phylogenetic analyses were performed with the complete S, M, and L segment sequences for Koum _2022 and the partial sequence of the L segment for Boki _2022. CCHFV strains were divided into several clades, which we labeled into genotypes based on the recent reclassification [10]. For both the L and S segments, our CCHF strains clustered with strains isolated in Mauritania in 1984 (ABB30015 and ABB30041), Spain in 2014 (ASV45882_SPN_2014 and ASV45880_SPN_2014), and Nigeria in 1996 and 1966 (AAY24690_NIG_1966 and ARB51456_NIG_1996_NIG) and other strains recently isolated in Matam, Senegal (Boki_CCHF_2019, ArD374334_SEN_2022, and ArD374517_SEN_2022) [12,13]. However, the genotypes of these two segments varied according to the genomic segments. Indeed, the typology of the L tree (Figure 2a) showed that the tick strain isolated in Koumpentoum (Koum_2022) and the human strain from Bokidiawe (Boki_2022) were very similar and belonged to genotype I, while the typology of the S tree showed that Koum_2022 belonged to genotype III (Figure 2c). Regarding the M segment, Koum_2022 also clustered within genotype I (Figure 2b). However, based on the M segment, this strain had the closest relationships with CCHF strains isolated from Russia (ABB30033_RUS_1985 and ABB30035_RUS_1985), Oman (ABB30032_OMN_1997), Pakistan (AAM48107_PAK_1976), and China (AAK52743_CHN).

**Figure 3:**
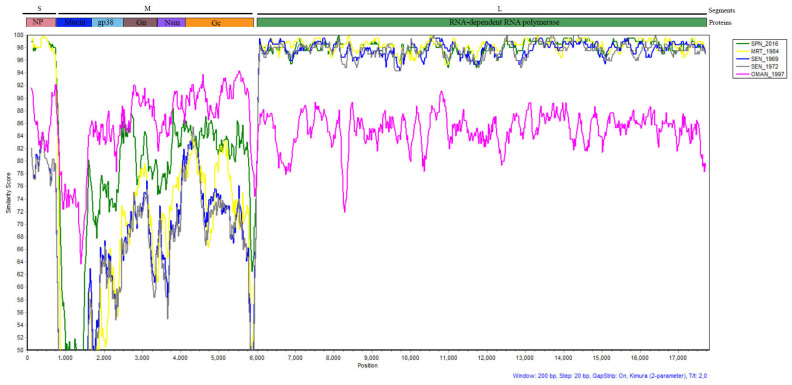
Similarity plot generated by SIMPLOT software version 3.5.1 based on the 3 genome segments. The tick strain (Koum_2022) was used as a query sequence, and isolates from Oman (OMAN_1997), Spain (SPN_2016), Senegal (SEN_1969 and SEN_1972), and Mauritania (MRT_1984) were used as representative parent sequences. The similarity score is expressed as percentage.

Considering the phylogenetic data of the three genome segments, the tick strain (Koum_2022) is thought to be a reassortant strain belonging to genotypes 1 and 3. The SIMPLOT analyses revealed high similarity (about 95–100%) between Koum_2022 and previous strains from Spain, Senegal, and Mauritania in the L segment, while higher genetic variabilities were observed in the S and M segments between these strains (Figure 3). Indeed, in the S segment, variations were observed along the genome, but Koum_2022 shared the highest similarities (97–100%) with strains from Mauritania and Spain compared to the old Senegalese strains. The M segment showed more genetic variability between all the strains compared to the other genome segments. These variations were detected along the genome segment but were more important in the Mucin region, where Koum-2022 showed only about 50% similarity with the other strains from Senegal, Mauritania, and Spain. Important variations were also observed between these strains in the gp38, Gc, and Gn regions (similarity ranging between 50% and 89% along the genome). Interestingly, the strain from Oman (OMAN_1997), which showed more distinctive features than the others strains in all genome segments, was the one that shared more similarities with our Koum_2022 strain in the M segment (similarity between 64 and 94% along the genome), especially in the Mucin region, where all other strains showed higher genetic variabilities (Figure 3).

**Figure 4:**
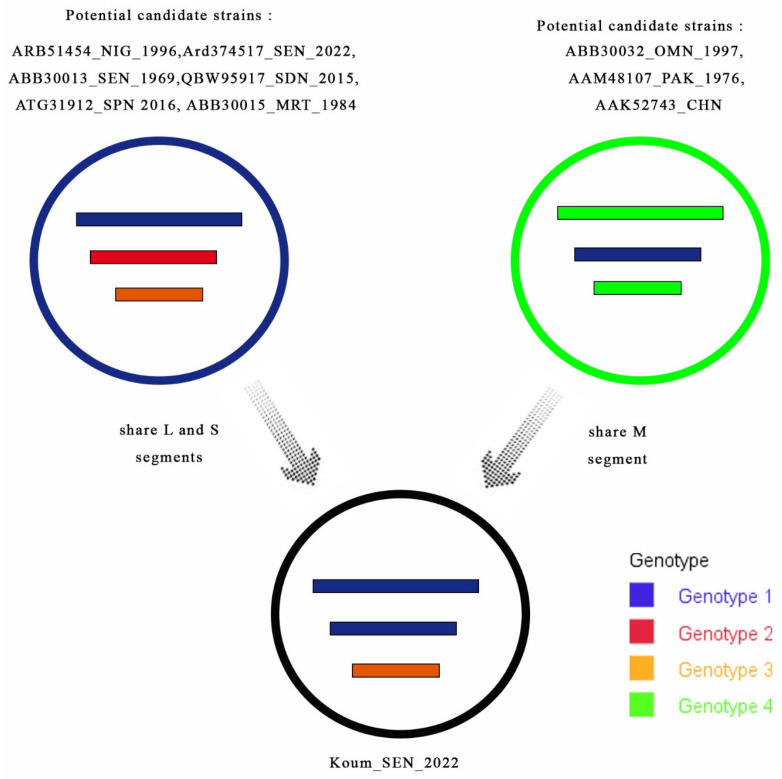
Scheme of genetic reassortment between CCHF genotypes. Each circle represents a different CCHF genotype. The 3 internal lines represent all genome segments according to their decreasing sizes (L, M, and S segments). The arrows indicate shared segments. The genotype of the tick strain (Koum_2022) was derived from reassortment events between the potential candidate strains.

Considering the phylogenetic and SIMPLOT data, the reassortment event of Koum_2022 seems to have occurred between different potential CCHF candidates belonging to multiple genotypes from different countries (Spain, Mauritania, and Oman) (Figure 4).

## 4. Discussion

In this study, we reported the detection of the CCHFV in humans in 2022 in Koumpentoum and Bokidiawe (Eastern Senegal) by two parallel and complementary surveillance systems. We also showed evidence of the circulation of the CCHFV in livestock (sheep and goats) in both areas as well as in *Hy. marginatum. rufipes* ticks collected during the outbreak investigations in Koumpentoum. Of the two human cases, only the one detected through 4S surveillance was positive using RT-PCR. Therefore, as already described for Rift Valley fever, our study also highlights the importance of this 4S surveillance system network for the early detection and confirmation of human cases, as it offers the possibility of genomic characterization of the circulating viral strains [15]. Sequencing and phylogenetic analyses of the CCHF strains isolated from the human case detected through the 4S network as well as ticks revealed high genetic variability between the CCHF strains circulating in Eastern Senegal. Indeed, our data showed the presence of a reassortant strain in ticks belonging to genotypes I and III and originating from CCHFV strains of varied origins and genotypes. The SIMPLOT analysis supported the findings of the phylogenetic trees and suggested that among all the CCHF strains analyzed in our study, the strains from Spain, Mauritania, and Oman were the most probable parental strains of Koum_2022. However, the level of similarity between the strain from Oman (OMAN_1997) and Koum_2022 on the M segment is lower than that observed between Koum_2022 and the strains from Spain and Mauritania on the S and L segments. This could be due to rapid genetic evolution of the M segment of the Koum_2022 strain after the reassortment event. Indeed, it has been shown that the M segment has greater genetic variation compared to the S and L segments, and this reflects the critical role of Gn and Gc glycoproteins in the viral life cycle [2]. This lowest similarity rate between the strain from Oman (OMAN_1997) and Koum_2022 could also be due to the fact that the parental strain for the M segment is another strain from Asia close to the Oman strain (OMAN_1997) for which we do not have complete genome sequences to include in our analyses. This once again shows the importance of the genomic characterization of strains in order to better understand the genetic diversity between strains as well as their phylodynamics and phylogeography. CCHFV isolates sharing the same features (multiple CCHF genotypes and probable reassortment) as our strain have already been described in Agnam Civol, another locality in the Matam region [13]. This supports the possible introduction of the CCHFV to Senegal from Mauritania and other countries as well as its spread inside the country through livestock exchanges and/or the migration of birds carrying infested ticks. Those ticks might be simultaneously infected by two virus strains with different evolutionary histories, which could lead to reassortment events [2]. These reassortments may increase the emergence risk of more virulent CCHF strains in Senegal.

Previous studies on the CCHFV in Northern Senegal (Saint-Louis region) have repeatedly demonstrated its circulation among humans, ruminants, and ticks [25]. Human CCHF cases have been reported in areas in Northern Senegal, in Rosso in 2021, and in Podor in 2022 as well as in the northeast regions, including Bokidiawe in September 2019 [12,26] and Agnam Civol in 2021 [13]. Our study contributes new data on CCHF detection in Bokidiawe and marks its first detection in the Tambacounda region (Koumpentoum) in the southeast of the country. Indeed, our data give new insights on the evolution of the CCHFV in Eastern Senegal and highlight its circulation in humans and *Hy. marginatum. rufipes* ticks, the principal vector of CCHF [14]. *Hy. marginatum. rufipes* ticks have the most CCHFV infection records among tick species (18%) [27]. In addition, the other tick species (*Hy. truncatum*) found in this area have previously been found to be associated with the virus in nature, potentially amplifying the risk of tick-to-human transmission [28,29,30,31]. Our study also revealed higher CCHFV seroprevalence in animals in the northeast, surpassing the overall mean seroprevalence of the CCHFV (24.6%) reported in a meta-analysis of 206 articles [32]. This finding confirms the high CCHF seroprevalence in livestock already described in Northern Senegal [33]. We also showed, for the first time, the detection of specific CCHF antibodies in animals in the southeast. These data suggest the permanent presence of the CCHFV infection in the northern region, at the border with Mauritania, and its diffusion to the southeast, probably through the movement of livestock. Indeed, a lot of animal movement occurs between Northern Senegal, which is a sylvo-pastoral area, and other regions in the country, and this could contribute to a large diffusion of CCHF in the country, with transhumant breeders being at high risk of CCHF exposure. Indeed, a serostudy of livestock and farm workers in South Africa revealed substantial occupational exposure amongst the latter, suggesting a CCHFV infection incidence five times higher than that reported in the literature. Similar serosurveys targeting cattle, sheep, goats (transhumant versus sentinel herds), and high-risk human groups (farm workers, breeders, veterinarians, butchers, etc.) versus low-risk groups (those with no exposure to livestock) are ongoing in different regions (northern, center, and southeast) to analyze the correlation between occupational exposure and CCHF seroprevalence in Senegal. This will help us better understand the transmission dynamics of CCHF in Senegal in order to implement control strategies.

Observations from remote sensing studies and long-term ground-based data collections indicate a positive trend in vegetation greenness and an increase in total herbaceous/woody foliage mass of 6%/20% in the Sahel [34,35]. The coincidence of climate change and the rural exodus in Senegal might impact the emergence of the CCHFV, similar to the situation observed in Turkey, where a rural exodus led to livestock practice preferences shifting from sheep to cattle, subsequently leading to observable vegetation changes [36,37].

Taken together, this study provides evidence of CCHF circulation in animals and ticks, substantial genetic diversity within the CCHFV, and escalating emergence in human populations. It suggests that Eastern Senegal, akin to Northern Senegal, represents a high-risk area for CCHF emergence. Consequently, it is crucial to bolster the epidemiological and zoological surveillance of the CCHFV using a sentinel approach to better understand its transmission dynamics and mitigate the risk of emergence.

## Figures and Tables

**Table 1:** CCHFV seroprevalence in goats and sheep in Bokidiawe and Koumpentoum.

Site	Species	Number of Samples	Number of CCHFV-Positive Samples (%)
	Sheep	73	27 (36.98)
Bokidiawe	Goats	22	16 (72.72)
	Total	95	43 (45.26)
	Sheep	18	2 (11.11)
Koumpentoum	Goats	57	2 (3.5)
	Total	75	7 (9.33)

**Table 2:** Ticks collected from livestock (sheeps, cattle, and goats) during investigations of human Crimean–Congo hemorrhagic fever cases in Eastern Senegal.

Species	Bokidiawe		Koumpentoum		Total	
	N	%	N	%	N	%
*Hyalomma impeltatum*	74	74.7	0	0.0	74	6.4
*Hyalomma marginatum rufipes*	2	2.0	344 *	32.6	346	30.0
*Hyalomma truncatum*	0	0.0	134	12.7	134	11.6
Others §	23	23.2	577	54.7	600	52.0
Total	99		1055		1154	

* one positive pool of four specimens; § others: *Amblyomma variegatum*, *Boophilus geigyi*, *Rhipicephalus eversi eversi*, *R. guilhoni*, *R. lunulatus*, and *R. sulcatus*.

## Data Availability

The metadata supporting the results of this study can be obtained by contacting the authors. Due to privacy concerns for research participants, particularly febrile patients, the data are not publicly accessible. The sequences of the L, M, and S segments from tick strains were submitted to Genbank with the following IDs: 2725049, 2725094, and 2725136. The human strain’s sequence was submitted to Genbank under the ID 2725143.
